# A High-Accuracy and Power-Efficient Self-Optimizing Wireless Water Level Monitoring IoT Device for Smart City

**DOI:** 10.3390/s21061936

**Published:** 2021-03-10

**Authors:** Tsun-Kuang Chi, Hsiao-Chi Chen, Shih-Lun Chen, Patricia Angela R. Abu

**Affiliations:** 1 Department of Electronic Engineering, Chung Yuan Christian University, Taoyuan City 320314, Taiwan; g10602603@cycu.edu.tw; 2 Department of Business Administration, Chung Yuan Christian University, Taoyuan City 320314, Taiwan; 3 Department of Information Systems and Computer Science, Ateneo de Manila University, Quezon City 1108, Philippines; pabu@ateneo.edu

**Keywords:** Internet of Things, smart city, power saving, self-optimizing, water level monitoring, self-adapting software engineering

## Abstract

In this paper, a novel self-optimizing water level monitoring methodology is proposed for smart city applications. Considering system maintenance, the efficiency of power consumption and accuracy will be important for Internet of Things (IoT) devices and systems. A multi-step measurement mechanism and power self-charging process are proposed in this study for improving the efficiency of a device for water level monitoring applications. The proposed methodology improved accuracy by 0.16–0.39% by moving the sensor to estimate the distance relative to different locations. Additional power is generated by executing a multi-step measurement while the power self-optimizing process used dynamically adjusts the settings to balance the current of charging and discharging. The battery level can efficiently go over 50% in a stable charging simulation. These methodologies were successfully implemented using an embedded control device, an ultrasonic sensor module, a LORA transmission module, and a stepper motor. According to the experimental results, the proposed multi-step methodology has the benefits of high accuracy and efficient power consumption for water level monitoring applications.

## 1. Introduction

With the continuous improvement of wireless devices, more and more wireless applications have been proposed to improve human life quality. To acquire and monitor human body signals for healthcare purposes, wireless body sensor network (WBSN) was proposed in [1]. The wireless sensors, or wireless devices, were strategically positioned to allow convenience and comfort in acquiring and monitoring the body signals. The smart city was defined by IBM to enhance the wider aspects of human living. Furthermore, a survey of the smart city concept and a description of its dimensions for impacting smart city development were discussed in [2]. The technology is the key factor of building a smart city with integrated software, hardware, and networks. The architecture of a smart city is composed of three layers, namely, perception, network, and application, which were illustrated in [3]. Information is transformed to digital data and is transmitted for storage and analysis. The analyzed results can be applied to various fields such as in transportation, health care, education, energy management, and environment, etc. 

In a sensible city, cost and energy usage are the major challenges in the development of smart city applications. The solutions of the Internet of Things (IoT) and the smart city was analyzed and discussed in [4], in which the solution should be replicable, scalable, and sustainable. Infrastructure and application services need to be modular and flexible, to adjust their scale to be widely used in different cities. The solution also needs to be designed with a cost-efficiency and power-efficiency that can keep system operations working under budget constraints. A survey of system running efficiency of device deployment, and a network management problem for a system was presented in [5]. As transmission distance impacts the energy consumption of a device, any node has to be deployed in an efficient way. To solve the set cover problem during deployment, a points of interests (POTs) set P and sensor node deployment set S were proposed in [5]. To satisfy the constraints, it provided a better approach, setting sensor nodes for device coverage, network connectivity, and network lifetime. Network lifetime is especially impacted by the trade-off between device power limitations and data accuracy. Data accuracy is a key factor in the data analysis of results in the application layer. To reduce the distance of transmission, a technique that optimizes the use of a drone to collect data from sensors was proposed in [6]. Apart from significantly reducing the power consumption through data gathering, the drone also provided a flexible way to handle complex situations in real time. 

In building a sensor network for IoT devices, PortoLivingLab was proposed in [7], which is an integration of the three monitoring platforms into a city-scale sensor system implemented in Porto. The platforms handled information of people, environments, and traffic flow. In PortoLivingLab, the devices are categorized into three types, depending on the sensing targets. A mobile phone is used for detecting human information like mood (by app) and location (by GPS). In environment sensing, the data collect unit (DCU) contains variable sensors to gather information from nature. Together with the processing unit and WIFI interface, the DCU transmits information to the backend for storage and analysis to improve living quality. On the other hand, in a vehicle platform, on-board units (OBUs) and road-side units (RSUs) are utilized to provide internet connection in the vehicle to enable tracking information of passengers as well as traffic flow. By integrating heterogeneous data sources, the IoT platform provides advance services like fuel consumption and map matching. In addition, the feasible solutions to implement the application based on IoT system were proposed in [8,9,10,11,12,13]. In these applications, multi-sensors were widely used to gather a wide variety of data. In [13], a different approach was proposed which utilizes multi-sensors to track targets with collaboration. In implementing collaboration between sensors, the sensors are activated alternatively to track the moving target. To improve the scheduling efficiency, adaptive sensor scheduling was proposed. The sensor that is activated to detect the target can use the information of the other available sensors to predict and select the next sensor to activate, by using an extended Kalman filter (EFK). This is a promising approach to reduce power efficiency and accuracy for periodic scheduling. For improving one-step prediction, in [14] the proposed multi-step sensor scheduling was implemented to predict the target trace and to schedule the next sensor to activate [15]. By calculating iteratively for prediction, the proposed methodology achieved a higher performance in target tracking in [15]. 

In the backend of a smart city framework, the quality of information is an important item to consider in evaluating the system quality. A framework for a smart city and discussion about information quality were presented in [16]. For the perception layer, accuracy was defined as correctness, precision, and completeness. To keep the quality of sensing data, the sensor or device should overcome the deviation in measurement, and solve the trade-off issue between power consumption and measurement resolution. Massive sensors are used in detecting variable data in real time in power grid applications [17]. For accurately dealing with energy data, the different sensors should be synchronized for data processing. In addition, phasor measurement units were proposed to implement advanced applications in a smart grid. To reduce the system cost of building a sensing network, sensors have to be smart and cost-efficiency. This means that the sensors with several functions, like data processing and data storage, and that are self-powered, will greatly help to keep the system running, and in reducing the maintenance cost. Therefore, an IoT or smart device in a sensing system is expected to be cost-efficient, power-efficient, and multi-functional. For efficiency, there were several studies that provided a significant cost-efficiency solution for device design. In wireless body sensor network (WSBN), a data reduction method was proposed for wireless devices in [18]. By using an adjacent signal, the device reduces the amount of data to improve power consumption during transmission. Several micro control unit (MCU) designs were presented in [19,20,21] to handle multiple data sources from different sensors. Sharing of control over the core was a brilliant way to reduce the cost of a sensor network. To enhance the system throughput, a new architecture and programming flow of ECG acquisition sensor systems in a power-efficient way were proposed in [22]. For adapting to different situations, Chen et al. proposed an adaptive fuzzy resolution control method to adjust the sampling rate of an analog-to-digital converter (ADC) in [23]. In this way, power consumption is efficiently reduced in low-level conditions. These studies provided outstanding contributions to wireless sensor networks. 

For improving the accuracy of the device or sensor, an additional resource is needed, such as power consumption or cost. It is difficult to balance the tradeoff between the accuracy and power consumption in the system. For a power grid system, Mousavi et al. used linear regression (LR) and support vector regression (SVR) prediction models to analyze prediction error, cost reduction, energy consumption, and training time in [24]. The proposed method found that SVR can improve prediction accuracy by 21% and save 16% of household power, but the energy consumption of SVR is much more than LR. By observing battery capacity, the SVR with the largest training set only uses 0.01% of 2000 mAh. The concern of energy consumption is the power budget in applications. Thus, the SVR model is preferred to improve accuracy and save costs for household power. In epilepsy monitoring, Imtiaz et al. considered detection sensitivity in the tradeoff between the accuracy and system power in [25]. For supporting diagnosis, the proposed method selected sufficient sensitivity to improve the power consumption.

In this paper, a multi-step measurement process was developed to improve the measurement precision in cost-efficiency and power-efficiency, and was realized on a smart device for water level monitoring. The device adjusted the sensor location to measure the water level by a multi-step method, and processed the raw data to acquire a more precise measurement result. Moreover, a self-optimizing power saving program is also proposed in this study. Generally, water level sensors are located outdoors. Hence, it is hard to acquire power for device operation. After installation, it is also challenging to replace the battery once exhausted. Thus, solar panel was included in this water level monitoring device to increase the power allocation. Under limited power allocation, a power management program is needed for balancing accuracy and power saving. A power management that was based on the power situation and measurement result provided the best power saving solution for the device operation, to keep both the accuracy and power efficient. 

## 2. Methodology

In this study, a multi-step measurement mechanism and power self-optimizing process were proposed and realized for water level monitoring applications. The multi-step measurement mechanism consists of a motor and a single sensor. The motor is used as the controller while the sensor, on the other hand, measures the target in different locations with different values. The measured results are corrected by the information of these locations and values to improve the accuracy. To increase the device lifetime in a smart city, a power self-optimizing process was proposed to schedule when the device will perform its transmission. By simulating the power conditions, the time of transmission is changed and the power efficiency is improved. These two methods improve the device efficiency to achieve the smart city requirements of cost-efficiency and power-efficiency. The details will be described in this section.

### 2.1. Multi-Step Measurement Mechanism

To be able to improve the measurement accuracy, previous studies widely used multiple sensors to measure the same targets, such as those in [26]. By processing the raw data through weight adjustment, the different sensors can efficiently get rid of the deviation of measurement. However, the increasing amount of sensors and complex calculations increase the cost of the devices for smart city applications. Table 1 shows the comparison of implementation between a single sensor and multiple sensors for accuracy improvement. Multiple sensors need more devices or sensors to be deployed in a defined area for obtaining raw data to improve the accuracy. Comparing device industrial design (ID) size, the device needs more space to place multiple sensors, which increases the cost. For maintenance, multiple sensors are more difficult to tune and will take more time to replace especially for a wider area of coverage. For the extendibility of the system, a single sensor is easier to implement by including additional devices or sensors. Therefore, a multi-step measurement process was proposed to improve the accuracy and keep the cost-efficiency. For distance measurement applications, the sensor is usually in a fixed location to measure the target to the required distance value. The deviation of the sensing value will decrease the accuracy of the measurement, which will affect the data analysis results in the smart city backend. In an ultrasonic distance sensor, Sahoo et al. provided an experimental result that lists the different required deviations given the different estimated distances in [27]. Using an ultrasonic sensor module, specifically the HC-SR04, the deviation of measurement was 0.8 cm at 100 cm, and 3.4 cm at 500 cm. Thus, a multi-step measurement mechanism was proposed to improve the deviation of measurement. 

**Figure 1:**
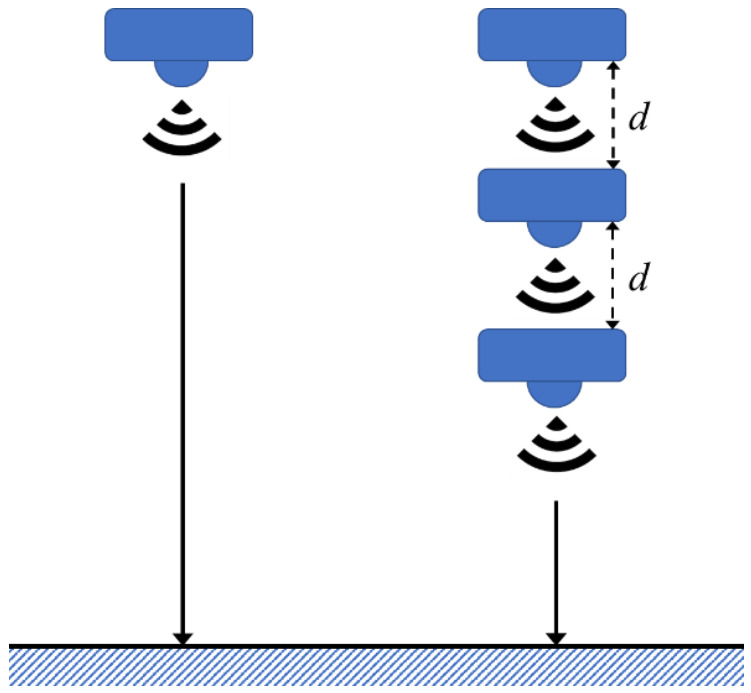
Single sensing and multi-step sensing diagram.

In the proposed mechanism, the device consists of a distance sensor and a motor. The concept of multi-step measurement uses a single sensor to measure the target in different locations. By processing the raw data, the single sensor only needs to process the deviation of a single sensor, instead of several deviations from multiple sensors. To decrease the deviation of measurement, data processing is utilized, which is of lower complexity for the single sensor system. After each measurement, the motor changes the sensor locations as shown in Figure 1. The sensing values and corresponding moving distances were acquired relative to both the sensor and the motor.

**Figure 2:**
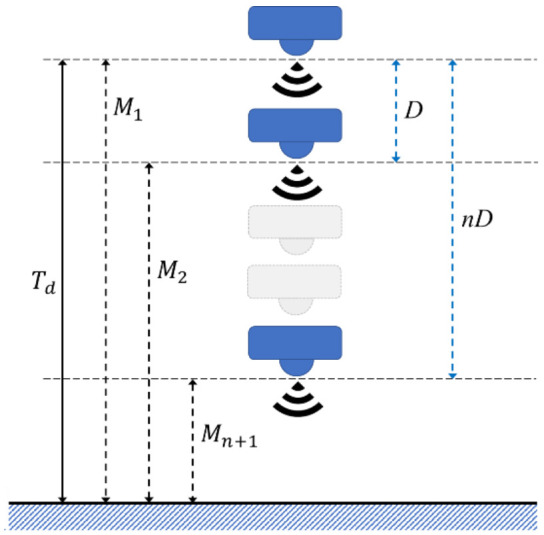
Distance naming rule in multi-step measurement.

In multiple-sensor measurement, raw data processing is designed for measuring the deviations of different sensors. A weighting rule was proposed in [26] to choose trusted data from sensors. In the single sensor system, raw data is only acquired from one sensor. The raw data processing has a lower complexity, as it handles data of one sensor only. In a multi-step measurement mechanism, the sensor measures the target actual distance $T_{d}$ with $M_{1}$ as the first location. Then, the motor moves the sensor by a fixed distance D to measure the target, and the sensor acquires the distance of the target as $M_{2}$ at another location. With n times movement, the mechanism acquires the distance data $M_{1}\ldots \;M_{n+1}$ with their corresponding moving distance D…nD, as shown in Figure 2. To improve the accuracy of measurement, an averaging method was proposed, and is presented in Equation (1). The total distances acquired in the measurement process are divided by the number of moves. By using this method, the device measures the target several times from different locations, and is therefore a multiple-sensor measurement in a cost-efficient way. As such, the number of sensors and complexity of raw data processing are reduced.

(1) $$T_{d}=\frac{M_{1}+(\sum_{k=1}^{n}M_{k+1}+kD)}{n+1}$$

### 2.2. Power Self-Optimizing Process

**Figure 3:**
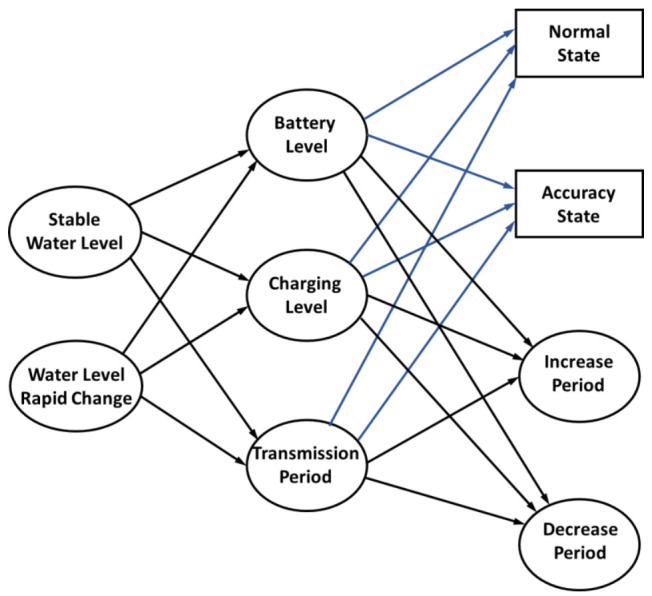
Decision making diagram of the proposed power self-optimizing process.

In a smart city application, there are many aspects that are considered to have an impact on the system maintenance, such as network management, device deployment, and device lifetime. Considering the large number of sensors or devices that are deployed in a sensing area to capture different information from the real world, improving the device lifetime is one effective way to increase system efficiency. In general, wireless devices rely on the use of a battery to provide power for data collection and transmission. Solar energy is widely used in IoT systems [28], which greatly improves power efficiency through self-charging. However, the consistently changing weather conditions are a challenge in continuously charging the devices. As illustrated in Section 2.1, although the motor improves the accuracy for data measurement, the additional power consumption decreases the lifetime of the system. Therefore, a power self-optimizing process is proposed to improve both the accuracy and power consumption of smart devices. Devices with a multi-step measurement mechanism exhibit two states, a normal state and an accuracy state. In the normal state, the device directly estimates the target distance. The result with device deviation is acquired in this state. As for the accuracy state, the moving sensor estimates the target multiple times and acquires a more accurate result through data processing. To save on the power consumption of the device, the wireless transmission period is also an important factor to consider. In an accuracy state, one measurement consumes a large amount of power for motor driving. If the transmission period is decreased, the number of measurements and power can be increased. The proposed process is illustrated in Figure 3. The power self-optimizing process will determine the state and transmission period for increasing the power efficiency.

To reduce flood damage and flexibly regulate the retarding basin, water level monitoring is an important application in a smart city sensor network. For these applications, the change of water level will be considered first in the proposed process. The change of water level is divided into two states or levels, the stable state and the rapid changing state. A previous study in [27] proposed a deviation for the actual estimated distance, wherein the deviation was less than 1% in the experiment. Thus, the difference of the current estimated water level and the last estimated water level over 1% implies that the water level is changing quickly. Otherwise, when the change is less than 1%, it implies that the water level is stable. Battery level, device charging level, and data transmission period are considered as power conditions in the device. With these power conditions, the device lifetime can be estimated. For example, the device transforms to a normal state and increases its transmission period to save on power consumption when the battery level and the charging level are low and the water level is stable. The power conditions of the device determine which state and which action is better. The main rule for improving power efficiency is that charging efficiency must be greater or equal to the total power consumption of the device, as shown in Equation (2). Equation (2) aims to keep the system current larger or equal to the charging current, which is used to maintain or charge the battery power. It is used to determine whether using multi-step measurement is possible or not, and to enable solar charging to cover the additional motor power consumption needed to avoid decreasing the device lifetime.

(2) $$P_{charge}\geq P_{total}$$

The water level monitoring device with multi-step measurement is composed of a micro-control unit, a transmission module, and a motor. Thus, the total power consumption ($P_{total}$) consists of the MCU power ($P_{mcu}$), the transmission module power ($P_{t}$), and the motor power ($P_{m}$), as shown in Equation (3). The power consumptions of the motor and the transmission module are affected by the transmission period. As the current sensor only provides instantaneous power, it is difficult to acquire the correct power consumption given the transmission period. Thus, the parameter *1/T* is added in Equation (3) to quantize power consumption with the transmission period *T*. The transmission period is the interval time of each multi-step measurement. The MCU power is continuous, but motor power is only produced in multi-step measurement. If the period is increased, the motor power and transmission module power are decreased. The ratio of motor power to system power is also decreased. Thus, the motor power and transmission power are divided by the transmission period. For example, if the transmission period is 10 s that means the motor and the transmission module would work once every 10 s. Thus, the parameter *1/T* is 1/10, which is the power ratio of the motor and transmission module which consumes 10% of the system power. Motor power depends on the transmission period and the device state. The state was defined to parameter q in Equation (4). When q=0, there is no additional motor power when the device is in normal state. Otherwise, the motor power is considered in $P_{total}$ when q=1. In the power self-optimizing process, the safety level is set by a constant to decide on the device mode. The safety level depends on solar irradiance and duration, and represents the charging level of the device. In this process, the safety level is used to select the mode of the device. When the battery level is greater or equal to the safety level, the device will turn to accuracy mode to increase the accuracy. The safety level is used to decide whether to use accuracy mode. The accuracy mode is affected by charging level, system power, and charging power. This can make the saving power strategy more defined. If the charging efficiency is high enough at the estimated location, the safety level can be adjusted to a lower level to get a higher accuracy. For example, the safety level was set to 80% in a 12 h simulation, the charging duration showed a 50% charging efficiency for 8 h, and 100% charging efficiency for 4 h. The details are mentioned in Section 3.2. For an abrupt change in water level, this implies that an emergency has occurred. The conditions will be ignored for that particular moment. The period will decrease to the minimum, and the measurement state will be forced to the normal state for measuring.

(3) $$P_{total}=\;P_{mcu}+\frac{1}{T}\;(P_{m}\times q\;+\;P_{t})$$

q=1, if battery level ≥ safety level

(4) q=0,     otherwise

The proposed method considered power conditions of the device to have a balance between charging and discharging states. Keeping the charging level over 60% ensures that the device is turned on and is operating overnight. In addition, to improve the power efficiency, the device tunes its transmission period, and decides the measurement state according to the above formulas to optimize the power consumption during its charging state. The device self-optimizes power consumption and accuracy by this method.

## 3. Experiment

### 3.1. Simulation of Multi-Step Measurement

In the experiment results in [27], the deviation was recorded for different estimated distances. The results showed that the deviation was proportional to the estimated distances and the percentage of deviation was maintained when the estimated distances increased. Table 2 lists each deviation for the different estimated distances. To verify the multi-step measurement mechanism, the sensing data were produced by deviation percentage, and then simulated by Matlab (Mathworks, Inc., Natick, MA 01760-2098 USA). For example, the percentage of deviation is 0.8% when the estimated distance is 100 cm in Table 2, and the sensing data were generated by −0.8% to +0.8% deviation, as per Equation (5).

(5) $$Sensing\;data=\;100+100\times random\left(-0.8\%\;to\;0.8\%\right)$$

**Figure 4:**
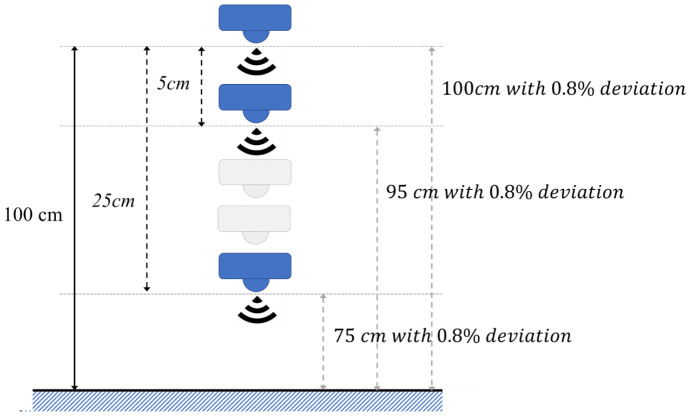
Simulation example when estimating distance is 100 cm.

Figure 4 shows an example when estimating distance is 100 cm. In this simulation, the moving distance D was set to 5 cm and the number of moves was set to five times. First, the sensor estimates the target at the default location as that of the result with the single-step measurement, then moving down and sensing repeatedly. After moving 5 times, the data will be processed following Equation (1) to acquire an estimated result. The data of the different locations were generated by a random function with a correlated deviation in one measurement. The simulation was repeated 1000 times to get an average estimated result in different estimating distances. Table 3 lists the simulation results of the multi-step measurement by deviation percentage of 1% to 5% in different estimated distances. Compared to the average result with the single-step measurement, the efficiency was improved by 0.014% at the 100 cm estimated distance, and by 0.006% at the 500 cm estimated distance. The percentage of deviation was less for long distances. Thus, the efficiency of the multi-step measurement is lower for longer distances. The proposed method improves the efficiency from 0.005% to 0.022% for 1% deviation. As for the different deviations in the same estimated distance, the efficiency was increased to 0.043% when the deviation was at 5%. The proposed multi-step measurement efficiently improved the accuracy when the deviation was larger. According to [27], the simulation results with related deviations are listed in Table 4. Table 4 lists the simulation results of multi-step measurement by [27], with experimental results of deviation percentages of 0.51% to 0.8%. The range of the efficiency was within 0.003% to 0.021% for a real ultrasonic sensor. Ignoring the single-step result with a larger error at the estimated distance 400 cm, the proposed multi-step measurement was shown to be more efficient for short estimated distances. For estimating the water level in a real world application, the deviation is unpredictable, because of environmental factors such as temperature and humidity. Both Table 3 and Table 4 show that the proposed multi-step measurement can efficiently reduce the deviation and improve the accuracy in a water level monitoring system application.

### 3.2. Efficiency of Power Self-Optimizing Process

In this paper, a power self-optimizing process was proposed to solve the additional power consumption of a multi-step measurement, and to improve the system power efficiency. To reduce the demand for power, solar panels are incorporated in smart city devices to make them self-charging. The ideal situation is that the solar panel can provide all the power needed by the system. To supply the additional power needed by the proposed multi-step measurement method, a power self-optimizing process is used by the device with a solar panel in order to tune the device setup and enable balancing the charging and discharging of current. Table 5 lists the hardware specifications of the system in which the battery capacity is 3000 mAh. This can support system operations for 24 h when the lowest current of device operation is at 125 mA. The stepper motor consumes 135 mA, and the motor startup current is 337.5 mA. The proposed multi-step method takes 10 s to move the sensor and perform the estimation. The method only works when the transmission period is set to over 10 s. In the proposed device, the transmission module uses about 1.26 mA, which is less than 0.5% of the whole system power consumption. Hence, it is small enough to ignore from the whole system power consumption. Thus, Equation (3) was simplified to Equation (6).

(6) $$P_{total}=\;P_{mcu}+\frac{1}{T}\;(P_{m}\times q)$$

To realize the method, the period of computation of the power self-optimizing algorithm is shown in the pseudocode below. The Algorithm 1 inputs include the battery level and charging current, and its output is the transmission period. The settings of the process include the default period, increasing level and decreasing level of period, maximum period, multi-step measurement operation time, and safety level. In the beginning, the next state is determined by the battery level. If the battery level is lower than the safety level, the proposed multi-step measurement function will be turned off to lower the power consumption. On the contrary, the proposed multi-step measurement function will be turned on to improve the accuracy. The total current of the system is calculated using Equation (6) for comparison with the charging power using Equation (2). The parameter *q* decides on adjusting the transmission time for the next operation period. Finally, in the next state, the charging current and total current are considered to decide on increasing or decreasing the period. If the multi-step measurement function is active, the period will increase to improve the power consumption, or decrease to improve the accuracy. As for the increasing period, the configuration of the maximum period, MaxPeriod, varies for different applications. In this simulation the maximum period is 120, for the longest estimated interval of 120 s. On the other hand, for the decreasing period, the limitation of the lowest period depends on the operating time of the multi-step measurement. With 5 cm and 5 times multi-step setups, the operation cost is about 10 s. As such, the multi-step operating time is 10 s for this simulation.
**Algorithm 1**: Period of computation of power self-optimizing process**Input:** Battery level, Charging current **Output:** Transmission period Period = 10; IncreaseLevel = 30; DecreaseLevel = 10; MaxPeriod = 120; MultistepOperationTime = 10; SafetyLevel = 80%; q = 1;**repeat**   **if** Battery level ≥ SafetyLevel **then**
*// select state according to battery level*   *NextState* = 1;   **else**   *NextState* = 0;   **endif**   $P_{total}=\;P_{mcu}+(P_{m}\times q)\;/\;\mathrm{Preiod}$; *// calculate total power*   **if**
*NextState* = 1 **then**
*// decide period*   **if**
$P_{total}>P_{charge}$
**then**     Period = Period + IncreaseLevel;     **if**
*Period* >= *MaxPeriod*
**then**     Period = MaxPeriod;     **endif**     **else**     Period = Period − DecreaseLevel;     **if**
*Period* < *MultistepOperationTime*
**then**     Period = MultistepOperationTime;     **endif**     **endif****endif**

**Figure 5:**
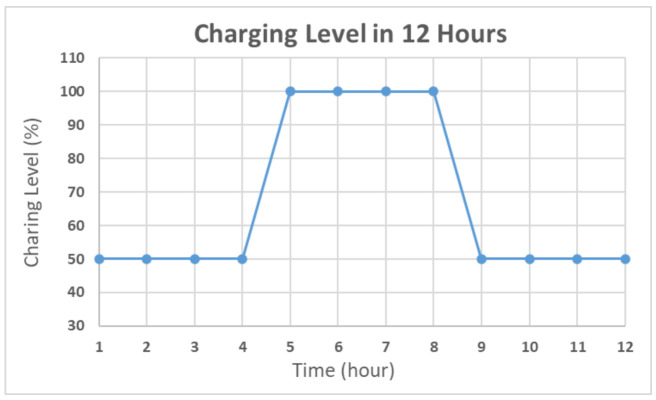
Charging level distribution over 12 h.

**Figure 6:**
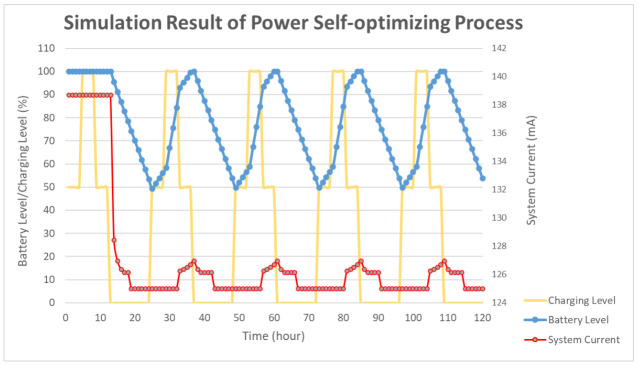
Simulation result for 120 working hours by the system operating for a period of 1 h.

**Figure 7:**
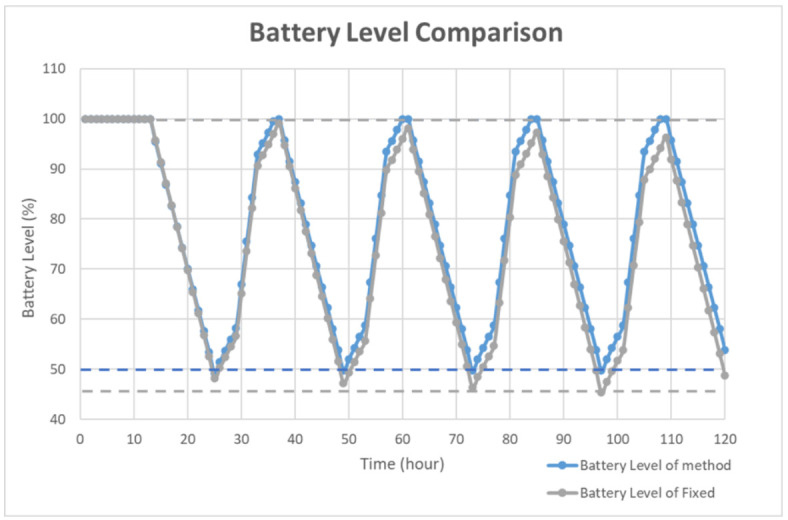
Battery level comparison of the proposed method and static operation.

To simulate the algorithm, the charging time was assumed to be 12 h. During the charging time, the charging level was set to 100% for 4 h, and 50% otherwise. The detailed distribution is shown in Figure 5. The other 12 h shows a 0% charging level for simulating the night. The battery level was set to 100% and the period was set to 10 s as the default. In the proposed simulation, the charging efficiency was assumed as ideal and simple for simulating the power optimizing process efficiency. In Figure 5, the solar duration was set to 12 h. The charging level was set to 100% for midday simulation and 50% for early morning and evening simulation. During the charging time, the charging level was set to 100% for 4 h and 50% for 8 h. The simulation showed that the average charging efficiency was 66.67%. The lowest power consumption of the device was 125 mA. Thus, the average efficient charging current was computed to be 131.68 mA. To set the safety level, the charging time was assumed as 6 h, half of the total charging duration. For a battery with 3000 mAh capacity, a 6 h charging with 131.68 mA could increase the battery level to about 26%. Taking the tolerance into consideration, the safety level was set to 80% in deciding when to use the multi-step measurement. The simulation results of the power self-optimizing process are shown in Figure 6, illustrating the charging level, battery level, and system current over time. The charging level was assumed to be 100% for 4 h, and 50% for 8 h for simulating the daytime. At night, the charging level was 0% for 12 h. The battery level was the system battery current capacity at each hour. According to the charging level, the power optimizing process has a tradeoff between the accuracy and system power consumption. For example, if the battery level is low and charging level is lower than the safety level, the process decreases system power by reducing the moving time of the sensor. The motor startup current is about 2.5 times the operation current, but it only happens for a moment. The startup current affects the system power but it has less effect by considering the sensor moving time of the multi-step measurement. In this implementation, the moving time was about 10 s. With an assumption of 0.1 s for the motor start up, the motor power was about 137.03 mA. For the first 15 h, the charging current could supply the whole system current requirement. The battery level stayed at 100%, and the multi-step measurement worked in its lowest period. After 12 h, the charging level decreased to 0. The battery level was drained without any charging current and the period was increasing to reduce the system power consumption. When the battery level was 80%, the multi-step measurement function was turned off to make the system operation in its lowest power consumption level at the 18th hour. At the 25th hour, the battery of the device started to get charged. The battery level increased gradually until it was 80% charged at the 32th hour to turn on the multi-step measurement. After which, the battery level changed between 50% to 100% due to the continuous power consumption and charging efficiency. By using this method, the multi-step measurement operated for 47.5% of the total simulation time, and the battery level was maintained at greater or equal to 50%. To compare with static operation, the simulation of the proposed method for a fixed period of 30 s is shown in Figure 7, with the battery level efficiently kept at 45%. However, the proposed method can also maintain its operation at 50% battery level. Thus, dynamically tuning the period can conserve the battery level, and keep it at a higher level. The efficiency of power is improved through the power self-optimizing process.

## 4. Experimental Results and Device Implementation

**Figure 8:**
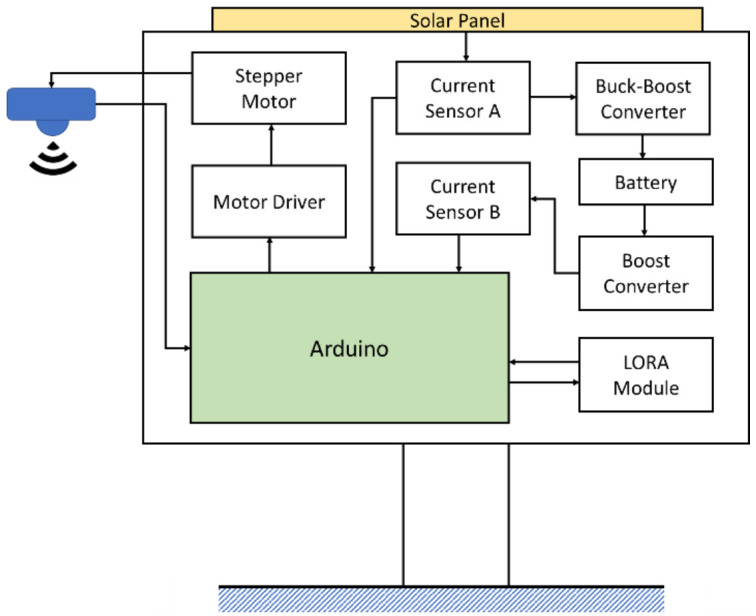
Block diagram of the proposed water level monitoring system.

**Figure 9:**
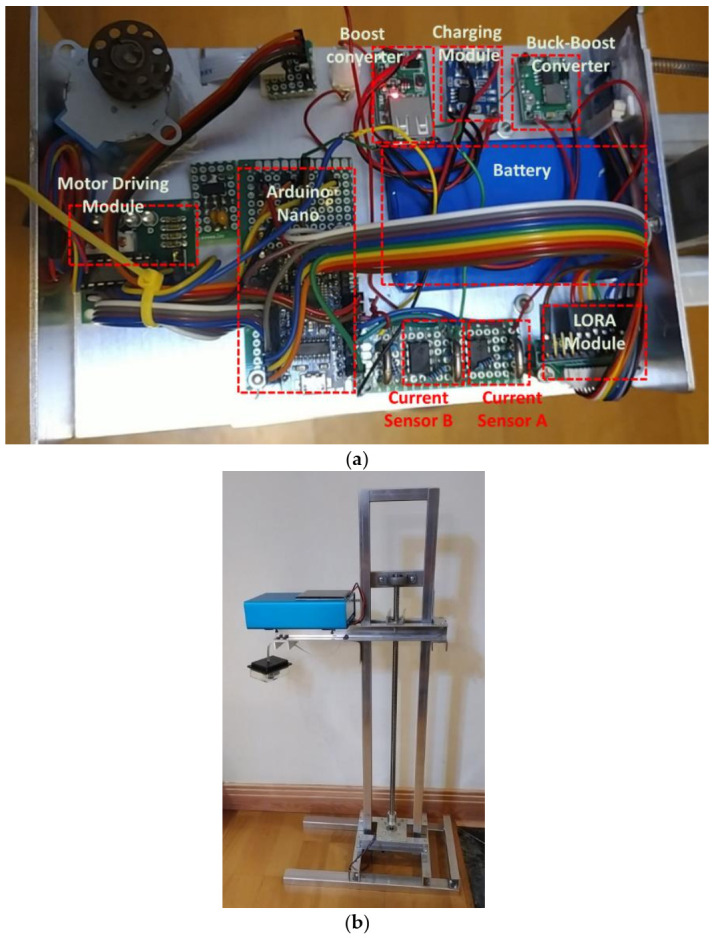
Realized water level monitoring system: (**a**) system structure, and (**b**) system appearance.

To improve on the power efficiency and accuracy, a multi-step measurement mechanism and power self-optimizing process are proposed in this paper. To implement the methodology, the system was built and designed for a water level sensing application. The system was composed of a solar panel, two buck converters, a battery, a LORA module, two current sensors, a stepper motor, a stepper motor driver, and an Arduino Nano as the control unit, as illustrated and shown in Figure 8 and Figure 9. For driving the stepper motor, the Arduino will send the control signal to the driver, then the ultrasonic sensor can move with the stepper motor and feedback the sensing data. In an accuracy state, the Arduino handles the raw data to acquire an estimated result. The LORA module transmits the estimated data to the backend whether in an accuracy state or in a normal state. In order to increase the efficiency of the device lifetime, a solar panel was designed and incorporated in the system to enable self-charging. When the solar panel transforms the solar energy to current, the current sensor A will detect the current to acquire the charging level. To transform the current to its charging level, the Arduino will calculate the current by converting the loss of 10%. To power up the Arduino, the battery discharging voltage needs to convert 5 V through another converter. The battery level impacts the power self-optimizing process. With that, the current sensor B estimates the current from the battery to acquire the battery level. The system was successfully designed and tested to measure the water level. The whole system was realized, with its circuit board and components illustrated in Figure 9. To acquire actual data, the setup of the multi-step measurement was moved at 3 cm five times, and moved at 5 cm five times. For multi-step measurement, the sensor starts at an initial location which is fixed. The motor will roll up the entire wire at each measurement to make sure that the initial location is in a consistent value every time. When the device turns to accuracy mode, MCU will send the information of direction and number of rotations to control the motor. The sensor will move a fixed distance according to a fixed number of rotations. Using the ultrasonic sensor module GY-US42V2, the estimated results are listed in Table 6. There were four different estimated distances used to test the efficiency of the multi-step measurement method. Referring to Table 6, the first row lists the estimated distance of 50 cm, the ultrasonic sensor estimated distance at the initial location of 50 cm. Then, the sensor was moved down by 3 cm using the proposed multi-step measurement to record the estimated water level and repeated 5 times. The recorded lowest estimated distance was 35 cm. To get the total measurement, the measurement was repeated 10 times and the average distance was computed for the different estimated distances. To estimate the efficiency of the multi-step measurement, the result using the single-step measurement was considered as the estimated data at the initial location, while the result using the multi-step measurement was considered as the estimated data processed using Equation (1). Results show that the highest efficiency was 0.39% at 200 cm, and the lowest efficiency was 0.16% at 50 cm. The proposed multi-step measurement was successfully implemented in hardware. Results show that it was able to efficiently improve the accuracy from the actual measurements acquired.

## 5. Conclusions

To improve the efficiency of IoT devices in a smart city application, this paper proposed a multi-step measurement mechanism and a power self-optimizing process. The multi-step method efficiently reduces the deviation of the sensor or estimating environment. By processing the raw data, the method provides a more accurate result for transmitting data to the backend of the smart city. In order to improve the accuracy using the multi-step measurement mechanism, the device needs additional power supply for continuous operation of the proposed method. A power self-optimizing process is proposed to monitor the balance between the accuracy and power consumption. Incorporating the use of a solar panel enables the device to extend its lifetime by self-charging. The proposed process calculates the charging level and system current in order to adaptively and dynamically adjust the settings for balancing the power. Compared with static estimation, the proposed process efficiently reduces the power consumption of the system. The system was realized using an Arduino Nano as the micro-control unit. The stepper motor and driving module with an ultrasonic sensor module (HC-SR04) were incorporated in the system to realize a multi-step measurement mechanism. The proposed methodology was successfully implemented with a high efficiency of accuracy and power, which allows the device to be used longer in a water level monitoring application, which is beneficial for smart city applications.

## Figures and Tables

**Table 1:** Comparison in terms of cost, size, maintenance, and scalability between a single sensor and multiple sensors.

	Single Sensor	Multiple Sensors
Cost	One device	N devices
ID size	Considering one sensor	Larger, considering multi-sensors
Maintenance	One device	Multiple devices
Scalability	High	Limited

**Table 2:** Maximum deviation of the estimated distances.

Estimated Distance (cm)	Maximum Deviation (cm)	Percentage
100	0.8	0.8%
150	1.2	0.8%
200	1.6	0.8%
350	1.8	0.51%
400	2.6	0.65%
500	3.4	0.68%

**Table 3:** Simulation results of multi-step measurement for deviation values of 1% to 5%.

Estimated Distance (cm)	Multi-Step Measurement Setup	Deviation	Result with Single-Step Measurement	Result with Multi-Step Measurement	Improved Efficiency of Accuracy
100	Move 5 cm 5 times	1%	100.01	100.00	0.014%
150		1%	150.03	150.00	0.021%
200		1%	200.02	200.01	0.006%
350		1%	349.97	349.99	0.005%
400		1%	400.08	399.99	0.022%
500		1%	499.96	499.99	0.006%
100		2%	99.98	100.00	0.015%
100		3%	100.03	99.99	0.035%
100		5%	100.10	100.05	0.043%

**Table 4:** Simulation results of multi-step measurement for deviation values of 0.51% to 0.8%.

Estimated Distance (cm)	Multi-Step Measurement Setup	Deviation	Result with Single-Step Measurement	Result with Multi-Step Measurement	Improved Efficiency of Accuracy
100	Move 5 cm 5 times	0.8%	99.98	99.99	0.015%
150		0.8%	150.03	150.00	0.021%
200		0.8%	200.03	200.02	0.003%
350		0.51%	349.99	350.00	0.003%
400		0.65%	399.95	400.03	0.021%
500		0.68%	500.03	500.01	0.005%

**Table 5:** System specification.

Items	Specification
Battery capacity	3000 mAh
Charging current	0~385 mA
Motor driving current	135 mA
Motor startup current	337.5 mA
System current	125 mA
System operating frequency	1 Hz
Transmission current	1.26 mA
Multi-step measurement operating time	10 s
Maximum transmission period	120 s

**Table 6:** Estimated results using the ultrasonic sensor module GY-US42V2.

Estimated Distance (cm)	Multi-Step Measurement Setup	Result with Single-Step Measurement	Result with Multi-Step Measurement	Improved Efficiency of Accuracy
50	Move 3 cm 5 times	49.8	49.88	0.16%
100		99.4	99.57	0.17%
150	Move 5 cm 5 times	149.7	150.16	0.31%
200		198.0	198.78	0.39%

## Data Availability

Not applicable.
